# Liver function indicators and risk of hepatocellular carcinoma: a bidirectional mendelian randomization study

**DOI:** 10.3389/fgene.2023.1260352

**Published:** 2024-01-22

**Authors:** Shanshan Qin, Jing Wang, Haiqing Yuan, Jingzhen He, Shoujing Luan, Yan Deng

**Affiliations:** ^1^ Department of Radiology, Qilu Hospital of Shandong University, Jinan, China; ^2^ Shandong Medical College, Jinan, China; ^3^ Department of Endocrinology and Metabolism, Weifang People’s Hospital, Weifang, Shandong, China; ^4^ Intensive Care Unit, Weifang People’s Hospital, Weifang, Shandong, China

**Keywords:** AST, ALT, GGT, ALP, hepatocellular carcinoma, mendelian randomization

## Abstract

Observational studies have shown an association between liver dysfunction and hepatocellular carcinoma (HCC), but the causality relationship between them is unclear. We aimed to determine whether there is a bidirectional causal relationship between liver function indicators (alanine aminotransferase, *ALT*; aspartate aminotransferase, *AST*; alkaline phosphatase, *ALP*; *γ*-glutamyltransferase, *GGT*) and HCC. Our two-sample Mendelian randomization (MR) study acquired single nucleotide polymorphisms (SNPs) associated with liver function indicators (*ALT*, *n* = 134,182; *AST*, *n* = 134,154; *GGT*, *n* = 118,309; *ALP*, *n* = 105,030) and with HCC (*n* = 197,611) from publicly available genome-wide association studies (GWAS) of East Asian ancestry in Japan (BioBank Japan, BBJ). Univariable MR analyses were performed to identify whether the genetic evidence of exposure was significantly associated with outcome. Multivariable MR analysis was conducted to estimate the independent effects of exposures on outcome. Univariable MR analysis indicated that the level of *ALT*, *AST*, and *GGT* was the risk factor for HCC incidence. Meanwhile, multivariable MR analysis revealed that *AST* was an independent risk factor for HCC. The hazard ratio (HR) of the probability of HCC was 3.045 [95% confidence interval (95%CI), 1.697–5.463, *p* = 0.003] for *AST*. The results of reverse MR analyses showed that gene-predictive HCC incidence could increase the levels of AST (HR = 1.031, 95%CI: 1.009–1.054, *p* = 2.52 × 10^−4^) and *ALT* (HR = 1.040, 95%CI: 1.019–1.063, *p* = 0.005). Meanwhile, HCC may be negatively correlated with *ALP* levels (HR = 0.971, 95%CI: 0.947–0.995, *p* = 0.018). This study provides evidence to support that genetically predicted higher levels of *AST* are related to increased risk of HCC, with no strong evidence of a causal effect of genetically predicted *ALP*, *ALP*, and *GGT* on HCC. In addition, genetic predisposition to HCC could influence blood concentration of *ALT*, *AST*, and *ALP*. Thus, this may create a vicious cycle.

## 1 Introduction

The burden of hepatocellular carcinoma (HCC) is an important healthcare problem and continues to be the most common histologic type of primary liver cancer (Toh et al., 2023). Japan has one of the highest rates of HCC in the world, with an estimated 34,000 HCC-related deaths in 2019 (Sung et al., 2021). The prevalence of HCC has also increased in recent years. In recent decades, considerable progress has been made in the study of the epidemiology, risk factors, molecular characteristics, and pathogenesis of HCC. Epidemiological and experimental studies have identified several major risk factors associated with hepatocarcinogenesis, including chronic hepatitis B/C, type 2 diabetes mellitus (T2DM), metabolic liver disease (particularly nonalcoholic fatty liver disease), and cirrhosis. Targeting these risk factors, therapeutic measures such as direct antivirals, and the use of metformin, are associated with risk reduction of HCC, and can even delay the postoperative recurrence of HCC (Wu et al., 2016; Tseng, 2018; Zhang et al., 2021). Identifying new risk factors and taking appropriate treatment measures will contribute to improving the prognosis of patients with HCC.

Serum liver enzymes, such as alanine aminotransferase (*ALT*), aspartate aminotransferase (*AST*), alkaline phosphatase (*ALP*), and *γ*-glutamyltransferase (*GGT*), are routinely measured clinical markers that represent different dimensions of liver dysfunction (Pratt and Kaplan, 2000). Physicians generally use significant elevations of liver enzyme levels as complementary markers to aid the diagnosis of various diseases. For example, elevations of *ALT* and *AST* may indicate the presence of hepatocellular predominant disorders while elevations of *ALP* and *GGT* may implicate cholestatic predominant diseases (Giannini et al., 2005). Epidemiological studies have shown the associations between abnormally high liver enzyme levels and risks and mortalities of many diseases, including HCC (Hann et al., 2012; Wu et al., 2022; Reddy et al., 2023). Several studies have shown that high *ALT* or *AST* levels are independent risk factors for the development of cirrhosis and HCC (Kawamura et al., 2012; Hernaez et al., 2013). Liver function abnormalities were also an independent prognostic indicator in patients with HCC (Zhang et al., 2019). Moreover, liver dysfunction may also affect the development of HCC in an indirect fashion (De Silva et al., 2019). Observational studies usually show that some liver function indicators, such as *ALT*, *AST*, *ALP*, and *GGT*, are associated with high risk of cardiovascular disease and type 2 diabetes, which are risk factors for HCC. Growing evidence shows that liver enzyme levels play important roles in HCC pathogenesis, such as tumorigenesis, local tumor progression, and metastasis. Due to the methodological limitations of traditional observational studies, including confounding and measurement error, these associations may be biased. Since the causal associations between liver function indicators and HCC risk have not been thoroughly investigated, identifying host factors predisposing individuals to HCC is urgently needed to improve primary prevention and develop treatment strategies.

Mendelian randomization (MR) is a method of examining the causal effect of a modifiable exposure to disease by using measured variation in genes of known function in observational data. Because the genotype of an individual is determined at conception and cannot be changed, there is no possibility of reverse causation or confounding bias being responsible for an association between genotype and disease (Davey Smith and Hemani, 2014). In recent years, many MR studies have emerged to provide clinical evidence (Chen et al., 2022; Liu et al., 2022; Pan et al., 2022). This proves that MR is a reliable research method to solve some problems, including finding risk factors for diseases.

We have used the largest available data sets to interrogate the potential effect of liver dysfunction, proxied by multiple biomarkers (*ALT*, *AST*, *ALP*, and *GGT*), on HCC risk. In addition, we have investigated whether HCC affects circulating liver function markers.

## 2 Methods

**FIGURE 1:**
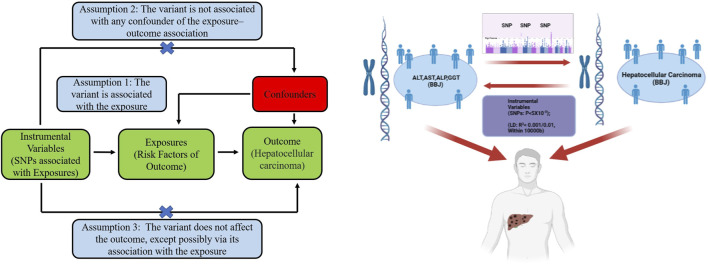
The three basic assumptions of Mendelian randomization (left) and the main design of this study (right). *ALT*: alanine aminotransferase; *AST*: aspartate aminotransferase; *ALP*: alkaline phosphatase; *GGT*: *γ*-glutamyltransferase. BBJ: Biobank Japan; LD: linkage disequilibrium; SNP: single nucleotide polymorphism.

We explored the relationship of four liver function markers (plasma concentration of *ALT*, *AST*, *ALP*, and *GGT*) with HCC. We also used MR to investigate whether predisposition to HCC is likely to have an impact on circulating *ALT*, *AST*, *ALP*, and *GGT*. The hypotheses, study design, and data sources used are detailed in Figure 1.

### 2.1 Summarized statistics of liver function indicators from a genome-wide association study in BioBank Japan

The exposure-related single nucleotide polymorphisms (SNPs) used in this study were obtained from the Biobank Japan Project (BBJ). BBJ started at the Institute of Medical Science, University of Tokyo, in 2003. To date, the BBJ Project has collected data on approximately 200,000 individuals with 47 different diseases. The genome-wide association studies (GWAS) summary statistics of liver function indicators were extracted from a study conducted by Masahiro Kanai (Kanai et al., 2018), who tested 5,961,600 autosomal variants and 147,353 X-chromosome variants for association with 58 traits in 162,255 Japanese individuals with East-Asian ancestry and identified 1,407 trait-associated loci (*p* < 5.0 × 10^−8^), 679 of which were novel. The GWAS summary statistics of liver function indicators in our study included 4 phenotypes: *ALT*, *AST*, *ALP,* and *GGT*. For *ALT* GWAS, the participants were 134,182 Japanese individuals. The GWAS summary statistics of *AST* and *GGT* comprised 134,154 and 118,309 Japanese individuals. For ALP GWAS, the study included 105,030 Japanese individuals. This study included 126,319 Japanese individuals and 6,108,953 SNPs. They focused on identifying different loci associated with liver function enzymes (Table 1).

**TABLE 1:** Summary of liver function and HCC.

Exposure	Number of SNP	Unit	Sample	R^2^	F	Consortium
*ALT*	25	NA	134182	3.25	57.30	Biobank Japan
*AST*	23	NA	134154	3.63	71.59	Biobank Japan
*GGT*	49	NA	118309	17.35	109.40	Biobank Japan
*ALP*	41	NA	105030	5.31	168.64	Biobank Japan
*HCC*	3	NA	197611	1.47	34.62	Biobank Japan

### 2.2 Extraction of SNPs associated with HCC

Summary-level statistical data for HCC were also obtained from a large GWAS of individuals with East-Asian ancestry in Japan. This study was conducted by Ishigaki et al. (2020) and aimed to address the problem that many participants in current genetic studies are of European ancestry. The study elucidated polygenic disease biology in the East Asian population by conducting a GWAS with 212,453 Japanese individuals across 42 disease traits. In this study, they adjusted for covariates including age, sex, and top five principal components (Table 1).

### 2.3 Mendelian randomization design and instrumental variables selection

MR is the use of genetic variants in non-experimental data to make causal inferences about the effect of an exposure on an outcome. In MR, genetic variant(s) are used as instrumental variables (IVs) for assessing the causal effect of the exposure on the outcome. The fundamental conditions for a genetic variant to satisfy to be an IV are as follows: 1) The IVs are associated with the exposures, 2) IVs are not associated with outcomes by means other than exposures, and 3) IVs cannot directly affect outcomes, if only through exposure. We selected the significant genetic variants associated with the exposures from GWAS (significant level *p* < 5 × 10^−8^). The minor allele frequency of the SNPs was >0.01. The SNPs used in our study were those that satisfied the linkage disequilibrium in the given genome region and the SNPs with palindromic structure were removed. When evaluating the causal relationship between liver function indicators and HCC, the threshold was *r* ^2^ < 0.001 and kb > 10,000. When evaluating reverse causality, the threshold was *r* ^2^ < 0.01 and kb > 10,000. For each variant included in the genetic instruments, variance (*R* ^2^) represents the variance in exposure explained by the genetic variant and was calculated using the formula *R* ^2^ = 2 × MAF × (1−MAF) × beta^2^ (where MAF represents the effect allele frequency and beta represents the effect estimate of the genetic variant in the exposure GWAS) (Palmer et al., 2012). F statistics (F = beta^2^/se^2^) were used to evaluate the remaining SNPs’ power. We calculated F statistics for each SNP. SNPs with F statistics <10 were identified to be weak instruments and we excluded them (Figure 1). The SNPs that were included in this analysis are listed in Supplementary Table S1.

### 2.4 Mendelian randomization analysis and sensitivity test

Inverse variance weighting (IVW) is a method of weighted average of random variables, where each random variable is weighted by the inverse of its variance. In this study, IVW was the main method adopted in the statistical analysis. Furthermore, the MR-Egger and weighted-median (WM) methods were used as supplements to the IVW method. For univariable MR, IVW, MR-Egger, and WM were used to estimate the effect of exposures on outcomes. For multivariable MR, regression-based IVW was used. The MR-PRESSO global test, outlier test, and distortion test were used to identify and remove SNPs with horizontal pleiotropy. If any outliers existed, we restarted an evaluation of the causal relationship. The intercept test of MR-Egger and Cochran’s Q test in the IVW and MR-Egger models were used to assess pleiotropy and the heterogeneity. In the case of pleiotropy, we preferred to use the MR-Egger. If the *p*-value in Cochran’s Q test was significant (*p* < 0.05), the WM model was used to analyze the statistics. Otherwise, a fixed-effects model was performed. Moreover, an online calculator was used to test the statistical power of this study (https://cnsgenomics.shinyapps.io/mRnd/). Genetic variants associated with exposures at genome-wide significance (*p* < 5 × 10^−8^) were then LD-pruned using the clump_data command in the “TwoSampleMR” package in R to identify an independent set of variants to serve as a genetic instrument for exposures. The univariable MR analysis was performed by R packages “Two Sample MR” and “Mendelian randomization”. The multivariable MR was performed by R packages “multivariable Mendelian randomization” (“MVMR”) and “Mendelian randomization”. The MR-PRESSO test was conducted using the R package “MRPRESSO”. Data visualization was conducted using R software 4.1.1 (https://www.r-project.org/).

## 3 Result

### 3.1 Causal effects of the liver function indicators on HCC

To investigate the causal effects of the liver function indicators on HCC, we constructed a genetic instrument for liver function indicators using 10–49 independent SNPs associated with the above five traits at a genome-wide level of significance (*p* < 5 × 10^−8^), which accounted for 1.00–17.35% of the variability in exposures. The mean F-statistic ranged from 34.62 to 168.64, which indicated that no weak instrument bias existed.

#### 3.1.1 Univariable MR analysis of exposures to HCC risks

In the univariable MR analysis stage, IVW was the main analysis method for MR. Our MR analysis indicated that there was strong evidence to support causality between higher levels of *ALT*, *AST,* and *GGT* with risk of HCC.

The hazard ratios of the probability of HCC were 1.890 (95% confidence interval (CI), 1.209-2.954, *p* = 0.005) for *ALT*, 2.909 (95%CI: 1.902-4.451, *p* = 8.55 × 10^−7^) for *AST*, 1.300 (95%CI: 1.048-1.611, *p* = 0.016) for *GGT*, and 0.908 (95%CI: 0.821–1.169, *p* = 0.818) for *ALP* (Table 2).

**TABLE 2:** The effect estimates, test of heterogeneity and test of pleiotropy of liver function on HCC.

Expo-sure	Number of SNP	MR methodology	Effect estimates HCC				Test of heterogeneity		Test of pleiotropy	
			OR	95%LCI	95%UCI	*p*-value	Cochrane Q test	Phetero-geneity	MR Egger intercept	Ppleio-tropy
*ALT*	25	IVW	1.890	1.209	2.954	0.005	53.795	4.450*10^−4^		
		MR-Egger	4.218	0.474	37.526	0.318	52.487	4.300*10^−4^	−0.029	0.456
		Weighted median	0.950	0.461	1.957	0.889				
*AST*	23	IVW	2.909	1.902	4.451	8.55*10^−7^	52.021	0.300*10^−4^		
		MR-Egger	16.547	2.552	107.304	0.007	44.179	0.002	−0.067	0.067
		Weighted median	1.452	0.680	3.101	0.335				
*GGT*	49	IVW	1.300	1.048	1.611	0.016	99.547	1.820*10^−5^		
		MR-Egger	0.965	0.529	1.761	0.908	96.912	2.54*10^−5^	0.0175	0.264
		Weighted median	1.114	0.756	1.644	0.586				
*ALP*	41	IVW	0.980	0.821	1.169	0.818	40.226	0.460		
		MR-Egger	1.091	0.793	1.502	0.595	39.579	0.444	−0.009	0.429
		Weighted median	0.984	0.755	1.283	0.908				

#### 3.1.2 Multivariable MR analysis of exposures to HCC risks

Furthermore, the causal relationship between liver function indicators and HCC was explored by conducting multivariable MR analysis. Among the four traits, we had observed that *ASP* had a causal effect on HCC occurrence when using SNPs-associated exposures. After the adjustment of other traits, *GGT* and *ALT* become non-significant. Multivariable MR analysis revealed that the hazard ratios of the probability of HCC were 3.045 (95%CI: 1.697-5.463, *p* = 2.77 × 10^−4^) for *AST*, 1.312 (95%CI: 0.713-2.414, *p* = 0.385) for *ALT*, 0.980 (95%CI: 0.0.779-1.232, *p* = 0.860) for *ALP*, and 1.296 (95%CI: 0.980-1.714, *p* = 0.072) for *GGT* (Table 3).

**TABLE 3:** The multivariable Mendelian randomization results of liver function and HCC.

Expo-sure	Number of SNP	Effect estimates HCC			
		OR	95%LCI	95%UCI	*p*-value
*ALT*	25	1.312	0.713	2.414	0.385
*AST*	23	3.045	1.697	5.463	0.003
*GGT*	49	1.300	0.980	1.714	0.718
*ALP*	41	0.980	0.778	1.232	0.385

### 3.2 Causal effects of HCC on liver function indicators

In order to explore the reverse causality between HCC and liver function indicators, we utilized the data from publicly available large-scale GWAS and deemed that genetically predicted HCC was associated with the levels of *ALT*, *AST,* and *ALP*. Specifically, HCC was associated with higher levels of *AST* and *ALT*. In contrast, HCC may have a causal relationship with lower levels of *ALP*. The MR effects of HCC on liver function indicators were: *ALT* (OR = 1.031, *p* = 0.005, 95%CI: 1.009-1.054), *AST* (OR = 1.040, *p* = 2.52 × 10^−4^, CI: 1.019-1.063), *ALP* (OR = 0.971, *p* = 0.018, CI: 0.947-0.995), and *GGT* (OR = 1.014, *p* = 0.242, CI: 0.991-1.038) (Table 4).

**TABLE 4:** The effect estimates, test of heterogeneity, and test of pleiotropy of HCC on liver function.

Outcome	SNPs	MR methodology	Effect estimates on liver function				Test of heterogeneity		Test of pleiotropy	
			OR	95%LCI	95%UCI	*p*-value	Cochrane Q test	Phetero-geneity	MR Egger intercept	Ppleio-tropy
*ALT*	rs113777417	IVW	1.031	1.009	1.054	0.005	2.600	0.273		
	rs7775228	MR-Egger	1.087	1.004	1.178	0.289	0.775	0.379	−0.013	0.406
	rs8107030	Weighted median	1.029	1.000	1.059	1.1048				
*AST*	rs113777417	IVW	1.040	1.019	1.063	2.52*10^−4^	1.364	0.506		
	rs7775228	MR-Egger	1.004	0.927	1.086	0.943	0.509	0.475	0.009	0.525
	rs8107030	Weighted median	1.035	1.007	1.064	0.013				
*GGT*	rs113777417	IVW	1.014	0.991	1.038	0.242	12.607	0.001		
	rs7775228	MR-Egger	1.134	0.924	1.391	0.441	5.625	0.017	−0.027	0.466
	rs8107030	Weighted median	1.022	0.982	1.064	0.286				
*ALP*	rs113777417	IVW	0.971	0.947	0.995	0.018	0.806	0.369		
	rs7775228	MR-Egger	1.049	0.956	1.150	0.498	3.714	0.156	−0.019	0.338
	rs8107030	Weighted median	0.971	0.941	1.001	0.061				

The effects between SNPs-associated exposures and outcomes were visualized using R software.

### 3.3 Sensitivity analysis

The pleiotropy of results was not tested in our study. MR-Egger intercept represented the average level of pleiotropy of all SNPs associated exposure. No significant horizontal pleiotropic effects were detected in the MR-Egger test (for the intercept of MR-Egger, all *p* values were more than 0.05). All the results of these exposures were MR-PRESSO-corrected results if outliers were detected. The statistical power of these exposures was 100%.

## 4 Discussion

HCC causes a heavy disease burden and is the fourth leading cause of cancer-related deaths worldwide (Siegel et al., 2023). Risk factors for the occurrence of HCC are numerous, including HBV and HCV infection, alcohol consumption, aflatoxin B1, and nonalcoholic fatty liver disease (Pan et al., 2022; Liu et al., 2023a; Pan et al., 2023). These conditions are associated with liver dysfunction and can lead to fibrosis, cirrhosis, and eventually HCC (Kotsiliti et al., 2023). Most studies exploring the risk factors for HCC development are based on observational studies and clinical experience. However, the major disadvantage of an observational study is that its validity is threatened by confounding by indication (De Nardi et al., 2022). Furthermore, studies have shown that genetic factors may also independently modulate HCC risk (Shimokawa et al., 2020). Human HCC genome sequencing studies have begun to uncover relationships between risk factors and mutated genes (Sun et al., 2021). MR studies use genetic variants as proxies of non-genetic risk factors to assess whether a risk factor is causally related to a disease. Although MR has already been used successfully in cancer epidemiology to estimate risk factors for overall cancer risk and cancer mortality, it has rarely been applied in the field of HCC study (Yarmolinsky et al., 2022; Wang et al., 2023).

Plasma concentrations of liver enzymes (*ALT*, *AST*, *ALP*, and *GGT*) are routinely measured clinical markers that represent different dimensions of liver dysfunction. *ALT*, located in the cytosol, and *AST,* located in the mitochondria, are released from damaged hepatic cells into the blood after hepatocellular injury or death (Song et al., 2012). *ALT* and *AST* are potentially useful surrogates for alcohol-induced liver disease and nonalcoholic fatty liver disease (NAFLD), defined as hepatic steatosis in the absence of excessive alcohol consumption (Kim et al., 2023). *ALP* is present in the ducts of the liver, and *GGT* is located on liver cell membranes (Inoue et al., 2023). The combined elevation of *ALP* and *GGT* can indicate obstructive or cholestatic liver disease, where bile is not properly transported from the liver because of an obstruction of the bile duct (Takahashi et al., 2023). *GGT* is also an indicator of alcohol use (De Silva et al., 2019). We conducted this bidirectional MR study to evaluate the potential causal effects between four liver function indicators (*ALT*, *AST*, *GGT,* and *ALP*) and HCC risk from a genetic perspective and to investigate whether predisposition to HCC might instead lead to liver dysfunction. Our findings from the MR analyses show evidence that genetic predisposition to higher circulating *AST* is related to higher risk of HCC. There was no strong evidence of a causal effect of genetically predicted *ALP*, *ALP* and *GGT* on HCC. In addition, genetic predisposition to HCC appeared to influence blood concentration of *ALT*, *AST,* and *ALP*. The present bidirectional MR study found that the main indicator of liver dysfunction (*AST*) increased the risk of HCC, suggesting that liver dysfunction exacerbates hepatocarcinogenesis and HCC could aggravate liver function damage. This may create a vicious cycle.

HCC patients often experience liver dysfunction, thus limiting the application of conventional therapies (Liu et al., 2023b). Therefore, it is particularly important to evaluate liver function in clinical practice. Nevertheless, the molecular mechanisms through which risk factors contribute to hepatocarcinogenesis, for the most part, remain poorly understood. Multiple studies have shown a direct role in liver function abnormalities in hepatic carcinogenesis (Kasprzak and Adamek, 2019). Several studies have shown that high *ALT* levels are an independent risk factor for the development of cirrhosis and HCC (Ogasawara et al., 2020; Dajti et al., 2021; Tahata et al., 2022). Liver function abnormalities were also an independent prognostic indicator in patients with HCC (Seong et al., 2022; Wong et al., 2023). Moreover, liver dysfunction may also affect the development of HCC in an indirect fashion. Observational studies usually show that some liver function indicators, such as *ALT*, *AST*, *ALP,* and *GGT*, are associated with a high risk of cardiovascular disease and type 2 diabetes, which are risk factors for HCC (Fard et al., 2022). Consequently, finding effective therapies for liver dysfunction in high-risk populations for HCC is a topic of long-standing interest and importance.

Our bidirectional MR provided comprehensive evidence to interrogate the potential effect of liver dysfunction on HCC risk. However, there are still some limitations in the present study. The limitations of available data hindered our ability to make strong conclusions about the potential association between liver dysfunction and HCC risk. First, because all the included data from GWAS used in this study were primarily focused on participants of East-Asian ancestry, there was bias against other ethnic groups with different lifestyles and cultural backgrounds. Second, all results were derived from genetic levels. There was a lack of prospective multicenter studies to confirm the causal relationship between liver dysfunction and HCC risk. Therefore, more studies are still needed to confirm our conclusions. Finally, although we used large-scale genetic data to obtain instrumental variables for our study, we did not manually check the validity of our instrument. However, we performed sensitivity analyses to assess horizontal pleiotropy and found that our results were robust to potential violations of this assumption.

## 5 Conclusion

This study provides a novel finding that individuals with East Asian ancestry who have higher genetic levels of *AST* are likely at risk of HCC. In addition, genetic predisposition to HCC could influence blood concentration of *ALT*, *AST,* and *ALP.* This may create a vicious cycle. Clinicians should raise awareness of *AST* in clinical practice.

## Data Availability

The original contributions presented in the study are included in the article/Supplementary Materials, further inquiries can be directed to the corresponding authors.

## References

[B1] Chen L. Yang H. Li H. He C. Yang L. Lv G. (2022). Insights into modifiable risk factors of cholelithiasis: a Mendelian randomization study. Hepatology 75 (4), 785–796. 10.1002/hep.32183 34624136 PMC9300195

[B2] Dajti E. Marasco G. Ravaioli F. Colecchia L. Ferrarese A. Festi D. (2021). Risk of hepatocellular carcinoma after HCV eradication: determining the role of portal hypertension by measuring spleen stiffness. JHEP Rep. 3 (3), 100289. 10.1016/j.jhepr.2021.100289 34095798 PMC8165428

[B3] Davey Smith G. Hemani G. (2014). Mendelian randomization: genetic anchors for causal inference in epidemiological studies. Hum. Mol. Genet. 23 (R1), R89–R98. 10.1093/hmg/ddu328 25064373 PMC4170722

[B4] De Nardi L. Simeone R. Torelli L. Maestro A. Zanon D. Barbi E. (2022). Pediatric males receiving hematopoietic stem cell transplant lose their male disadvantage in disease risk after the procedure: a retrospective observational study. Int. J. Cancer 151 (2), 191–199. 10.1002/ijc.33978 35195275 PMC9314096

[B5] De Silva N. M. G. Borges M. C. Hingorani A. D. Engmann J. Shah T. Zhang X. (2019). Liver function and risk of type 2 diabetes: bidirectional mendelian randomization study. Diabetes 68 (8), 1681–1691. 10.2337/db18-1048 31088856 PMC7011195

[B6] Fard M. T. Najafi F. Rezaeian S. Kohsari M. Moradinazar M. (2022). Association between serum liver enzymes and hypertension using propensity score matching analysis: evidence from a large Kurdish prospective cohort study. BMC Cardiovasc Disord. 22 (1), 476. 10.1186/s12872-022-02884-3 36357838 PMC9647908

[B7] Giannini E. G. Testa R. Savarino V. (2005). Liver enzyme alteration: a guide for clinicians. Cmaj 172 (3), 367–379. 10.1503/cmaj.1040752 15684121 PMC545762

[B8] Hann H. W. Wan S. Myers R. E. Hann R. S. Xing J. Chen B. (2012). Comprehensive analysis of common serum liver enzymes as prospective predictors of hepatocellular carcinoma in HBV patients. PLoS One 7 (10), e47687. 10.1371/journal.pone.0047687 23112834 PMC3480412

[B9] Hernaez R. Yeh H. C. Lazo M. Chung H. M. Hamilton J. P. Koteish A. (2013). Elevated ALT and GGT predict all-cause mortality and hepatocellular carcinoma in Taiwanese male: a case-cohort study. Hepatol. Int. 7 (4), 1040–1049. 10.1007/s12072-013-9476-6 26202033

[B10] Inoue K. Fujita R. Nagahara T. Murakami S. Nagai Y. Moriwake R. (2023). Predictive factors for recovery from alcoholic liver failure. Acta Med. Okayama 77 (2), 169–177. 10.18926/AMO/65146 37094954

[B11] Ishigaki K. Akiyama M. Kanai M. Takahashi A. Kawakami E. Sugishita H. (2020). Large-scale genome-wide association study in a Japanese population identifies novel susceptibility loci across different diseases. Nat. Genet. 52 (7), 669–679. 10.1038/s41588-020-0640-3 32514122 PMC7968075

[B12] Kanai M. Akiyama M. Takahashi A. Matoba N. Momozawa Y. Ikeda M. (2018). Genetic analysis of quantitative traits in the Japanese population links cell types to complex human diseases. Nat. Genet. 50 (3), 390–400. 10.1038/s41588-018-0047-6 29403010

[B13] Kasprzak A. Adamek A. (2019). Mucins: the old, the new and the promising factors in hepatobiliary carcinogenesis. Int. J. Mol. Sci. 20 (6), 1288. 10.3390/ijms20061288 30875782 PMC6471604

[B14] Kawamura Y. Arase Y. Ikeda K. Seko Y. Imai N. Hosaka T. (2012). Large-scale long-term follow-up study of Japanese patients with non-alcoholic Fatty liver disease for the onset of hepatocellular carcinoma. Am. J. Gastroenterol. 107 (2), 253–261. 10.1038/ajg.2011.327 22008893

[B15] Kim B. K. Bergstrom J. Loomba R. Tamaki N. Izumi N. Nakajima A. (2023). Magnetic resonance Elastography-Based prediction model for hepatic decompensation in NAFLD; a Multi-Center cohort study. Hepatology 78, 1858–1866. 10.1097/HEP.0000000000000470 37203233 PMC10663382

[B16] Kotsiliti E. Leone V. Schuehle S. Govaere O. Li H. Wolf M. J. (2023). Intestinal B-cells license metabolic T-cell activation in NASH microbiota/antigen-independently and contribute to fibrosis by IgA-FcR signalling. J. Hepatol. 79, 296–313. 10.1016/j.jhep.2023.04.037 37224925 PMC10360918

[B17] Liu H. Han C. L. Tian B. W. Ding Z. N. Yang Y. F. (2023a). Tenofovir versus entecavir on the prognosis of hepatitis B virus-related hepatocellular carcinoma: a systematic review and meta-analysis. Expert Rev. Gastroenterol. Hepatol. 17, 623–633. 10.1080/17474124.2023.2212161 37148261

[B18] Liu H. Yang C. C. Ma Y. L. Yang Y. F. Yan L. J. Ding Z. N. (2023b). Identification of the most effective subgroup of advanced hepatocellular carcinoma from immune checkpoint blocker treatment: a meta-analysis. Immunotherapy 15 (9), 669–678. 10.2217/imt-2022-0114 37140011

[B19] Liu Y. Xu H. Zhao Z. Dong Y. Wang X. Niu J. (2022). No evidence for a causal link between *Helicobacter pylori* infection and nonalcoholic fatty liver disease: a bidirectional Mendelian randomization study. Front. Microbiol. 13, 1018322. 10.3389/fmicb.2022.1018322 36406444 PMC9669663

[B20] Ogasawara N. Saitoh S. Akuta N. Sezaki H. Suzuki F. Fujiyama S. (2020). Advantage of liver stiffness measurement before and after direct-acting antiviral therapy to predict hepatocellular carcinoma and exacerbation of esophageal varices in chronic hepatitis C. Hepatol. Res. 50 (4), 426–438. 10.1111/hepr.13467 31785120

[B21] Palmer T. M. Lawlor D. A. Harbord R. M. Sheehan N. A. Tobias J. H. Timpson N. J. (2012). Using multiple genetic variants as instrumental variables for modifiable risk factors. Stat. Methods Med. Res. 21 (3), 223–242. 10.1177/0962280210394459 21216802 PMC3917707

[B22] Pan G. Q. Jiao Y. Meng G. X. Dong Z. R. Li T. (2023). The relationship between the serum lipid profile and hepatocellular carcinoma in east Asian population: a mendelian randomization study. Heliyon 9 (6), e17126. 10.1016/j.heliyon.2023.e17126 37484252 PMC10361312

[B23] Pan G. Q. Yang C. C. Shang X. L. Dong Z. R. Li T. (2022). The causal relationship between white blood cell counts and hepatocellular carcinoma: a Mendelian randomization study. Eur. J. Med. Res. 27 (1), 278. 10.1186/s40001-022-00900-y 36471350 PMC9724280

[B24] Pratt D. S. Kaplan M. M. (2000). Evaluation of abnormal liver-enzyme results in asymptomatic patients. N. Engl. J. Med. 342 (17), 1266–1271. 10.1056/NEJM200004273421707 10781624

[B25] Reddy K. R. McLerran D. Marsh T. Parikh N. Roberts L. R. Schwartz M. (2023). Incidence and risk factors for hepatocellular carcinoma in cirrhosis: the multicenter hepatocellular carcinoma early detection strategy (HEDS) study. Gastroenterology 165 (4), 1053–1063.e6. 10.1053/j.gastro.2023.06.027 37429366 PMC10529044

[B26] Seong G. Sinn D. H. Kang W. Gwak G. Y. Choi M. S. Lee J. H. (2022). Age and fibrosis index for the prediction of hepatocellular carcinoma risk in patients with high hepatitis B virus DNA but normal alanine aminotransferase. Eur. J. Gastroenterol. Hepatol. 34 (1), 69–75. 10.1097/MEG.0000000000001915 32925504

[B27] Shimokawa M. Yoshizumi T. Itoh S. Iseda N. Sakata K. Yugawa K. (2020). Modulation of Nqo1 activity intercepts anoikis resistance and reduces metastatic potential of hepatocellular carcinoma. Cancer Sci. 111 (4), 1228–1240. 10.1111/cas.14320 31968140 PMC7156873

[B28] Siegel R. L. Miller K. D. Wagle N. S. Jemal A. (2023). Cancer statistics, 2023. CA Cancer J. Clin. 73 (1), 17–48. 10.3322/caac.21763 36633525

[B29] Song M. Schuschke D. A. Zhou Z. Chen T. Pierce W. M. Wang R. (2012). High fructose feeding induces copper deficiency in Sprague-Dawley rats: a novel mechanism for obesity related fatty liver. J. Hepatol. 56 (2), 433–440. 10.1016/j.jhep.2011.05.030 21781943 PMC3261305

[B30] Sun Y. Wu L. Zhong Y. Zhou K. Hou Y. Wang Z. (2021). Single-cell landscape of the ecosystem in early-relapse hepatocellular carcinoma. Cell 184 (2), 404–421.e16. 10.1016/j.cell.2020.11.041 33357445

[B31] Sung H. Ferlay J. Siegel R. L. Laversanne M. Soerjomataram I. Jemal A. (2021). Global cancer statistics 2020: GLOBOCAN estimates of incidence and mortality worldwide for 36 cancers in 185 countries. CA Cancer J. Clin. 71 (3), 209–249. 10.3322/caac.21660 33538338

[B32] Tahata Y. Sakamori R. Yamada R. Kodama T. Hikita H. Nozaki Y. (2022). Risk of hepatocellular carcinoma after sustained virologic response in hepatitis C virus patients without advanced liver fibrosis. Hepatol. Res. 52 (10), 824–832. 10.1111/hepr.13806 35749289

[B33] Takahashi Y. Seko Y. Yamaguchi K. Takeuchi K. Yano K. Kataoka S. (2023). Gamma-glutamyl transferase predicts pemafibrate treatment response in non-alcoholic fatty liver disease. J. Gastroenterol. Hepatol. 38, 1743–1749. 10.1111/jgh.16222 37221601

[B34] Toh M. R. Wong E. Y. T. Wong S. H. Ng A. W. T. Loo L. H. Chow P. K. H. (2023). Global epidemiology and genetics of hepatocellular carcinoma. Gastroenterology 164 (5), 766–782. 10.1053/j.gastro.2023.01.033 36738977

[B35] Tseng C. H. (2018). Metformin and risk of hepatocellular carcinoma in patients with type 2 diabetes. Liver Int. 38 (11), 2018–2027. 10.1111/liv.13872 29956875

[B36] Wang Z. Lu J. Hu J. (2023). Association between antihypertensive drugs and hepatocellular carcinoma: a trans-ancestry and drug-target Mendelian randomization study. Liver Int. 43 (6), 1320–1331. 10.1111/liv.15566 37005366

[B37] Wong Y. J. Nguyen V. H. Yang H. I. Li J. Le M. H. Wu W. J. (2023). Impact of fatty liver on long-term outcomes in chronic hepatitis B: a systematic review and matched analysis of individual patient data meta-analysis. Clin. Mol. Hepatol. 29, 705–720. 10.3350/cmh.2023.0004 37157776 PMC10366810

[B38] Wu C. K. Chang K. C. Hung C. H. Tseng P. L. Lu S. N. Chen C. H. (2016). Dynamic α-fetoprotein, platelets and AST-to-platelet ratio index predict hepatocellular carcinoma in chronic hepatitis C patients with sustained virological response after antiviral therapy. J. Antimicrob. Chemother. 71 (7), 1943–1947. 10.1093/jac/dkw097 27073265

[B39] Wu H. C. Jeng W. J. Pan M. H. Hsieh Y. C. Lu S. N. Chen C. J. (2022). Incidence of hepatocellular carcinoma in a community-based Taiwanese population without chronic HBV/HCV infection. JHEP Rep. 4 (2), 100410. 10.1016/j.jhepr.2021.100410 35079699 PMC8777288

[B40] Yarmolinsky J. Díez-Obrero V. Richardson T. G. Pigeyre M. Sjaarda J. Paré G. (2022). Genetically proxied therapeutic inhibition of antihypertensive drug targets and risk of common cancers: a mendelian randomization analysis. PLoS Med. 19 (2), e1003897. 10.1371/journal.pmed.1003897 35113855 PMC8812899

[B41] Zhang L. X. Lv Y. Xu A. M. Wang H. Z. (2019). The prognostic significance of serum gamma-glutamyltransferase levels and AST/ALT in primary hepatic carcinoma. BMC Cancer 19 (1), 841. 10.1186/s12885-019-6011-8 31455253 PMC6712845

[B42] Zhang Y. Wang H. Xiao H. (2021). Metformin actions on the liver: protection mechanisms emerging in hepatocytes and immune cells against NASH-related HCC. Int. J. Mol. Sci. 22 (9), 5016. 10.3390/ijms22095016 34065108 PMC8126028

